# Oncological Safety of Skipping Axillary Lymph Node Dissection in Patients with Clinical N0, Sentinel Node-Positive Breast Cancer Undergoing Total Mastectomy

**DOI:** 10.1245/s10434-024-15049-7

**Published:** 2024-02-17

**Authors:** Jung Whan Chun, Eunhye Kang, Hong-Kyu Kim, Han-Byoel Lee, Hyeong-Gon Moon, Jong Won Lee, Wonshik Han

**Affiliations:** 1 Division of Breast Surgery, Department of Surgery, Seoul National University College of Medicine, Seoul, Republic of Korea; 2 Division of Breast Surgery, Department of Surgery, University of Ulsan College of Medicine, Asan Medical Center, Seoul, Republic of Korea; 3 Department of Surgery and Cancer Research Institute, Seoul National University College of Medicine, Seoul, Republic of Korea

**Keywords:** Breast cancer, Total mastectomy, Axillary lymph node dissection, Sentinel lymph node biopsy, Radiation therapy

## Abstract

**Objective:**

This study aimed to determine whether sentinel lymph node biopsy (SLNB) alone could afford oncological outcomes comparable with axillary lymph node dissection (ALND) in patients with early breast cancer without palpable lymphadenopathy who underwent total mastectomy (TM) and were SLN-positive.

**Methods:**

This study analyzed clinical data of 6747 patients with breast cancer who underwent TM between 2014 and 2018 in two tertiary hospitals in Korea. Overall, 643 clinical stage T1-3 N0 patients who did not receive neoadjuvant therapy and had one to two metastatic SLNs at the time of surgery were included. Propensity score matching was performed between the SLNB alone and ALND groups, adjusting for clinical *T* stage and number of metastatic SLNs. In total, 237 patients were allocated to each group.

**Results:**

Mean number of metastatic SLNs was 1.2 for the SLNB group and 1.6 for the ALND group. With a median follow-up of 65.0 months, 5 year disease-free survival was 90.8% for the SLNB group and 93.9% for the ALND group (hazard ratio [HR] 1.35, 95% confidence interval [CI] 0.70–2.58; *p *= 0.36). 5 year ipsilateral locoregional recurrence-free survival (LRRFS) was not significantly different between the two groups (95.1% and 98.3% for the SLNB and ALND groups, respectively) [HR 1.86, 95% CI 0.69–5.04; *p *= 0.21]. In the SLNB group, patients who received radiation therapy (RT) showed superior 5 year LRRFS than patients who did not receive RT (100% vs. 92.9%; *p *= 0.02).

**Conclusion:**

Collectively, our findings suggest that SLNB could afford comparable outcomes to ALND in patients with early breast cancer and one to two metastatic SLNs who underwent TM. Importantly, RT could decrease locoregional recurrence in patients who underwent SLNB alone.

**Supplementary Information:**

The online version contains supplementary material available at 10.1245/s10434-024-15049-7.

The presence of metastatic sentinel lymph nodes (SLNs) is one of the most important factors governing treatment decisions in patients with breast cancer. Following the American College of Surgeons Oncology Group (ACOSOG) Z0011 trial, surgery for breast cancer has trended toward less invasive axillary surgery for patients presenting limited metastatic SLN involvement.^1–3^ According to the Z0011 trial, survival results of patients without axillary lymph node dissection (ALND) were non-inferior to those of patients who underwent ALND. Notably, the Z0011 trial included patients with one to two SLNs who received breast-conserving surgery, whole-breast irradiation, and adjuvant systemic treatment. The National Comprehensive Cancer Network guidelines have adapted these results to omit ALND for patients with SLN metastases meeting the same criteria.^4^

It is widely accepted that a less invasive strategy for axillary nodal management can spare a subset of patients from potentially harmful and persistent morbidity associated with ALND. Compared with sentinel lymph node biopsy (SLNB) alone, ALND has been associated with complications, including lymphedema of the affected arm, wound infection, axillary seroma, and paresthesia.^5^ Approximately 75% of patients who undergo ALND reportedly exhibit at least one complication, in contrast to 25% of those who undergo SLNB alone. Thus, avoiding short- and long-term adverse effects of ALND in appropriate candidates could substantially impact the quality of life of patients.

Axillary radiation therapy (RT) has been evaluated in several studies to determine whether it could produce comparable effects to ALND. The AMAROS trial prospectively compared ALND and axillary RT in patients with T1–T2 primary breast cancer without palpable lymphadenopathy and SLN metastases.^6^ The authors reported that ALND or axillary radiation could afford effective and comparable regional control in terms of the 5 year axillary recurrence rate. Furthermore, disease-free survival (DFS) and overall survival (OS) did not differ substantially between the two groups; however, the difference in the rate of arm lymphedema was statistically significant in favor of axillary RT. Patients who underwent total mastectomy (TM) comprised 18% of the eligible cohort in the AMAROS trial. Accordingly, the omission of ALND has gradually increased in patients with SLNs undergoing TM in recent years.^7,8^ However, the oncologic safety of skipping ALND in patients undergoing TM remains debatable, given that the AMAROS trial was underpowered and included a limited proportion of patients who underwent TM.^9^

In the present study, we aimed to assess whether SLNB alone could afford comparable oncological outcomes to ALND in patients with SLN-positive early breast cancer without palpable lymphadenopathy who underwent TM. In addition, we evaluated the effect of adjuvant RT in this cohort.

## Methods

### Study Population and Cohort Selection

We reviewed the medical records of 6737 patients with cT1-3, cN0, M0 primary breast cancer who underwent TM with SLNB between January 2014 and December 2018 at the Seoul National University Hospital (SNUH) [*N* = 2695] or Asan Medical Center (AMC) [*N* = 4042]. Herein, we included patients with documented clinical N0 status based on physical examination, radiologic assessment, and pathologic evaluation if needed. According to the final pathology assessment, all patients had one to three metastatic SLNs. All patients with regular follow-up records available for at least 1 year were included, however we excluded patients with bilateral breast cancer, who received neoadjuvant chemotherapy, and whose preoperative diagnosis was ductal carcinoma *in situ*. In addition, patients with pregnancy-associated breast cancer and who were diagnosed with other types of malignancies during the follow-up period were excluded.

Finally, 643 patients from both institutions were included for further data analysis. The median follow-up period post-surgery was 65.0 (range 12–101) months. Patients were evaluated on an outpatient basis for disease recurrence according to standard practice guidelines, which included biannual or annual patient history, physical examination, and imaging studies. Considering the retrospective nature of this analysis, as well as to avoid selection bias, we performed 1:1 propensity score matching (PSM) between the SLNB alone and ALND groups, adjusting for the clinical *T* stage and the number of metastatic SLNs. Accordingly, 237 patients were included in each group for further analysis.

### Sentinel Lymph Node Detection Methods

To identify SLNs, we utilized 99 m Tc-sulfur colloid that was diluted in normal saline for radiopharmaceutical agent to detect with a gamma probe (NeoProbe2000, US Surgical, Norwalk, CT, USA). We injected 1 cc of the agent periareolarly and applied a gentle massage for 5 min. Furthermore, we used indigo carmine when available to localize SLNs as part of the dual mapping method. Along with the most intensely radioactive nodes, clinically enlarged, firm, or palpable axillary lymph nodes without active gamma signal were also excised and were included in the total number of SLNs.

## Outcomes

DFS, locoregional recurrence-free survival (LRRFS), and distant metastasis-free survival (DMFS) were defined as the interval from surgery to disease recurrence at any site; to disease recurrence at the chest wall, skin, internal mammary nodes or axillary nodes; and to disease relapse at a distant site, respectively. For subgroup analysis considering RT, we first grouped patients according to whether or not they underwent RT, and further divided the subgroup based on the extent of axillary surgery, to compare the DFS. Second, among patients who underwent SLNB alone, we compared the LRRFS and DMFS rates between subgroups, with or without adjuvant RT.

The pathologic stage classification was based on definitions presented in the 8th edition of the American Joint Committee on Cancer staging system. The present study was approved by the Institutional Review Board of SNUH (H-2302-090-1405) and AMC (20231341), Seoul, South Korea. Because the study was based on retrospective clinical data with minimal risk research, the need for informed consent was waived by the above-mentioned institutions.

### Statistical Analysis

PSM between the two groups was performed using variables such as the initial clinical T stage and number of positive SLNs. We utilized the Pearson Chi-square test and conditional logistic regression to compare detailed variables between the two groups. The DFS, LRRFS, and DMFS were estimated using the Kaplan–Meier method, and the results were compared using the log-rank test. All reported *p* values were two-sided and statistical significance was set at *p* < 0.05. Statistical analyses were performed using *R* version 3.1.0 (The *R* Foundation for Statistical Computing, Vienna, Austria) and SPSS Statistics version 23.0 (IBM Corporation, Armonk, NY, USA).

## Results

### Baseline Characteristics

In total, 643 patients were identified as eligible for TM for primary invasive breast cancer with one to three metastatic SLNs for analysis. Of these patients, 237 underwent SLNB alone, while 406 underwent completion ALND. Electronic supplementary material (ESM) e Table 1 summarizes the clinicopathologic characteristics of the total cohort. We utilized two clinical variables, i.e., clinical *T* stage and number of metastatic SLNs, for 1:1 PSM, with 237 patients in each group. Table 1 summarizes the demographic and pathologic characteristics of the matched cohorts. The ALND group tended to exhibit a higher number of total metastatic nodes with pathologic N stage and was more likely to experience lymphovascular invasion and receive adjuvant chemotherapy than the SLN-alone group. The proportion of patients with a human epidermal growth factor receptor 2 (HER2)-positive result was 17.3% for the SLNB-alone group and 22.7% for the ALND group. The patients were treated with adjuvant trastuzumab intravenously every 3 weeks for a total of 18 cycles.

**Table 1:** Baseline clinicopathologic characteristics of patients who underwent SLNB or ALND after propensity score matching

	SLNB alone (%)	ALND (%)	*p*-Value
Number of patients	237	237	
Age, years	52.9 ± 11.6	51.6 ± 9.5	0.18
			0.64
≤50	107 (45.1)	112 (47.3)	
>50	130 (54.9)	125 (52.7)	
Follow-up period, months	63.9 ± 18.6	65.2 ± 18.2	0.44
Clinical *T* stage			1
1	57 (24.1)	57 (24.1)	
2	145 (61.2)	145 (61.2)	
3	35 (14.8)	35 (14.8)	
Number of metastatic sentinel nodes			1
1	208 (87.8)	208 (87.8)	
2	26 (11.0)	26 (11.0)	
3	3 (1.3)	3 (1.3)	
Number of total axillary nodes	7.9 ± 5.2	17.2 ± 5.8	<0.001
Number of total metastatic nodes	1.2 ± 0.6	1.6 ± 1.3	<0.001
Pathologic *T* stage			0.21
1	88 (37.1)	75 (31.6)	
2	139 (58.6)	142 (59.9)	
3	9 (3.8)	17 (7.2)	
4	1 (0.4)	3 (1.3)	
Pathologic *N* stage			0.03
1	232 (97.9)	223 (94.1)	
2	3 (1.3)	13 (5.5)	
3	2 (0.8)	1 (0.4)	
Biologic subtype			0.3
ER+/HER2−	181 (76.4)	168 (70.9)	
ER+/HER2+	27 (11.4)	29 (12.2)	
ER−/HER2+	14 (5.9)	25 (10.5)	
ER−/HER2−	15 (6.3)	15 (6.3)	
Histologic grade			0.11
1	14 (5.9)	20 (8.4)	
2	168 (70.9)	147 (62.0)	
3	55 (23.2)	70 (29.5)	
Lymphovascular invasion			0.02
Yes	105 (44.3)	129 (54.4)	
No	132 (55.7)	108 (45.6)	
Adjuvant chemotherapy			0.001
Yes	182 (76.8)	210 (88.6)	
No	55 (23.2)	27 (11.4)	
Adjuvant radiation			0.36
Yes	72 (30.4)	63 (26.6)	
No	165 (69.6)	174 (73.4)	
Adjuvant endocrine therapy			0.2
Yes	205 (86.5)	195 (82.3)	
No	32 (13.5)	42 (17.7)	
*Recurrence*			
Locoregional recurrence			0.21
Yes	11 (4.6)	6 (2.5)	
No	226 (95.4)	231 (97.5)	
Distant metastasis			1
Yes	15 (6.3)	15 (6.3)	
No	222 (93.7)	222 (93.7)	

*ALND* axillary node dissection, *SLNB* sentinel lymph node biopsy, *ER* estrogen receptor, *HER2* human epidermal growth factor receptor 2

We further categorized the matching cohort of patients who underwent SLNB alone according to whether or not adjuvant RT was performed, and subsequently compared the results. Table 2 presents the baseline characteristics for these patient subgroups. Patients who received RT tended to have higher pathologic T and N stages than those who did not undergo RT.

**Table 2:** Clinicopathologic characteristics of patients with or without adjuvant RT among the matched cohort of those who underwent SLNB alone

	RT yes (%)	RT no (%)	*p*-Value
Number of patients	72	165	
Age, years			0.67
≤50	34 (47.2)	73 (44.2)	
>50	38 (52.8)	92 (55.8)	
Clinical *T* stage			0.21
1	12 (16.7)	45 (27.3)	
2	48 (66.7)	97 (55.8)	
3	12 (16.7)	23 (13.9)	
Number of metastatic sentinel nodes			0.02
1	62 (86.1)	146 (88.5)	
2	7 (9.7)	19 (11.5)	
3	3 (4.2)	0 (0.0)	
Number of total axillary nodes	7.1 ± 4.6	8.3 ± 5.4	0.08
Number of total metastatic nodes	1.3 ± 0.8	1.1 ± 0.4	0.01
Pathologic *T* stage			<0.001
1	15 (20.8)	73 (44.2)	
2	48 (66.7)	91 (55.2)	
3	8 (11.1)	1 (0.6)	
4	1 (1.4)	0 (0.0)	
Pathologic *N* stage			0.003
1	67 (93.1)	165 (100.0)	
2	3 (4.2)	0 (0.0)	
3	2 (2.8)	0 (0.0)	
Biologic subtype			0.91
ER+/HER2−	55 (76.4)	126 (76.4)	
ER+/HER2+	7 (9.7)	20 (12.1)	
ER−/HER2+	5 (6.9)	9 (5.5)	
ER−/HER2−	5 (6.9)	10 (6.1)	
Adjuvant chemotherapy			0.21
Yes	59 (81.9)	123 (74.5)	
No	13 (18.1)	42 (25.5)	
Adjuvant endocrine therapy			0.67
Yes	143 (86.7)	148 (85.1)	
No	22 (13.3)	26 (14.9)	
*Recurrence*			
Locoregional recurrence			0.02
Yes	0 (0.0)	11 (6.7)	
No	72 (100.0)	154 (93.3)	
Distant metastasis			
Yes	3 (4.2)	12 (7.3)	0.36
No	69 (95.8)	153 (92.7)	

*RT* radiation therapy, *SLNB* sentinel lymph node biopsy, *ER* estrogen receptor, *HER2* human epidermal growth factor receptor

Table 3 provides details regarding RT performed at both institutions, including irradiation field, radiation dose and fraction, and number of patients in each hospital. The median total radiation dose was 45.9 Gy (minimum, 40.5; maximum, 59.4), and the median number of fractions was 17.0 (minimum, 15; maximum, 33). Table 4 summarizes information regarding patients who developed locoregional recurrence with or without RT among all matched cohorts of patients.

**Table 3:** Radiation therapy location, dose, and fraction of all patients

Location	Gy	Fraction	Number of patients	Hospital SNUH/AMC
Chest wall	45.9–48	17–20	12	12/0
	50.4	28	5	5/0
Chest wall, axilla	43.2	16	1	1/0
Chest wall, SCL	43.2–55.2	16–20	27	27/0
	50–50.4	25–28	6	2/4
Chest wall, IMN	50	25	1	0/1
Chest wall, axilla, SCL	40.5–48.6	15–20	16	16/0
	50–59.4	25–33	44	11/33
Chest wall, SCL, IMN	43.2–52.8	16–20	3	3/0
Chest wall, axilla, SCL, IMN	43.2–54.4	16–20	71	71/0
	50–50.4	25–28	10	2/8
Total	Median (45.9)^a^	Median (17)^a^	196	150/46

^*^SCL supraclavicular area, *IMN* internal mammary node, *SNUH* Seoul National University Hospital, *AMC* Asan Medical Center

^a^Radiation dose: median 45.9 (minimum 40.5, maximum 59.4); radiation fraction: median 17.0 (minimum 15, maximum 33)

**Table 4:** Details of locoregional recurrence sites with or without radiation therapy among all matched cohorts of patients

Locoregional recurrence site	Number of patients
Axillary nodes (ALN)	7
Supraclavicular nodes (SCLN)	1
Internal mammary nodes (IMN)	3
ALN, SCLN	1
Chest wall	13
Chest wall, ALN	1
Chest wall, ALN, SCLN	1
Total	27
Radiation therapy	Number of patients
Yes	1^a^
No	26

^a^The radiation field for this patient was the chest wall and supraclavicular area, and the recurrence site for this patient was the internal mammary node

### Survival Outcomes

**Fig. 1:**
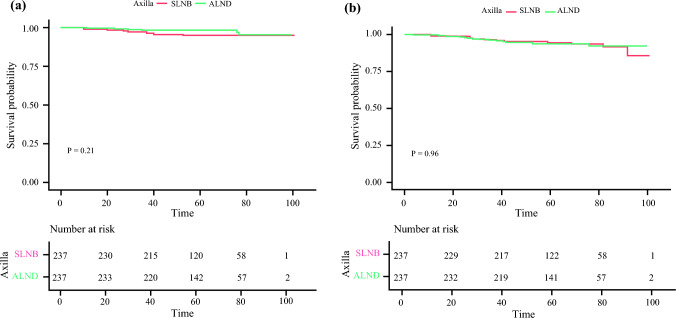
Survival analysis after propensity score matching to compare the SLNB-alone and ALND groups. The Kaplan–Meier method was used to analyze (**a**) locoregional recurrence-free survival and (**b**) distant metastasis-free survival. *ALND* axillary node dissection, *SLNB* sentinel lymph node biopsy

The 5 year LRRFS did not significantly differ between the two groups (95.1% and 98.3% in the SLNB-alone and ALND groups, respectively) [hazard ratio (HR) 1.86, 95% confidence interval (CI) 0.69–5.04; *p* = 0.21] (Fig. 1a). Furthermore, the 5 year DMFS did not significantly differ between the two groups (94.5% and 93.8% in the SLNB-alone and ALND groups, respectively) [HR 1.01, 95% CI 0.49–2.08; *p* = 0.96] (Fig. 1b).

**Fig. 2:**
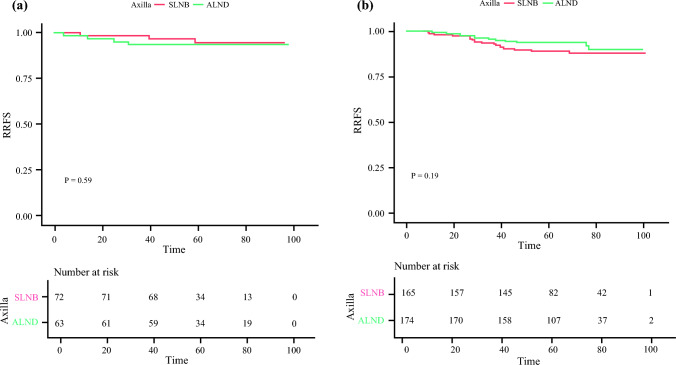
Five year disease-free survival between the SLNB-alone and ALND groups. Within the matched cohort, the 5 year disease-free survival was compared between the SLNB-alone and ALND groups, considering patients treated (**a**) with or (**b**) without radiation therapy. *ALND* axillary node dissection, *SLNB* sentinel lymph node biopsy, *RRFS* regional recurrence-free survival

Among patients who underwent RT, DFS rates were 94.5 and 93.7% in SLNB and ALND groups, respectively (HR 1.51, 95% CI 0.33–6.76; *p* = 0.59) [Fig. 2a]. Considering the subgroup without RT, DFS rates were 89.1 and 94.0% in the SLNB and ALND groups, respectively (HR 0.61, 95% CI 0.29–1.27; *p* = 0.19) [Fig. 2b]. ESM e Tables 2 and 3 summarize the clinical and pathological data of these patients within the matched cohorts.

**Fig. 3:**
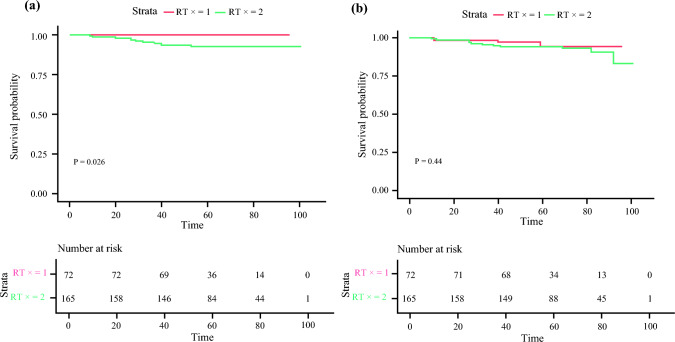
Five year locoregional recurrence-free survival and distant metastasis-free survival rates of the SLNB-alone group. Among patients who were treated with SLNB alone, the 5 year (**a**) locoregional recurrence-free survival and (**b**) distant metastasis-free survival were compared according to radiation therapy. (RTx = 1, with radiation therapy; RTx = 2, without radiation therapy). *SLNB* sentinel lymph node biopsy

Among patients who underwent SLNB alone, those who received RT tended to have higher pathologic T and N stages than those who did not undergo RT (Table 2); however, patients who received RT had a better 5 year LRRFS than those who did not undergo RT (100% and 92.9% for RT and no RT, respectively; *p* = 0.02) [Fig. 3a]. The 5 year DMFS rates were 94.5 and 94.4% in patients who received RT and those who did not undergo RT, respectively (*p* = 0.44) [Fig. 3b]. Among patients who underwent ALND, the 5 year LRRFS and DMFS did not differ significantly according to whether or not the patients underwent RT (LRRFS: 100 and 97.6%, *p* = 0.12; DMFS: 93.7 and 93.8%, *p* = 0.99, with or without RT, respectively); these data are provided in ESM e Fig. 1.

According to Table 4, most (26/27) patients who developed locoregional recurrence did not receive RT. Considering the one patient who underwent RT and experienced locoregional recurrence, the radiation field was the chest wall and supraclavicular area, while recurrence occurred in the internal mammary node.

## Discussion

A less invasive but effective surgical strategy for axilla has been an evolving area of interest in breast cancer surgery. Although traditional ALND has a firm foundation with proven efficacy for local control, the effectiveness of SLNB alone with or without RT for patients with clinically node-negative early breast cancer, who are candidates for TM, remains uncertain. Moreover, the effectiveness of post-mastectomy radiation in this setting warrants further validation.^7,10,11^

In the present study, less extensive axillary surgery with SLNB alone afforded regional control and survival rates that were comparable with those in response to ALND in patients with clinical N0 status early-stage breast cancer and one to two metastatic SLNs who underwent TM. There were no statistically significant differences between the SLNB-alone and ALND groups in terms of 5 year regional recurrence, distant metastasis, and DFS. Similar patterns were observed within the RT-treated and -untreated groups. Nonetheless, within the SLNB-alone group, there was a significant difference in locoregional recurrence, favoring patients who underwent adjuvant RT; however, patients who underwent RT experienced relatively more unfavorable clinical features than those who did not undergo RT. Accordingly, our results suggest that RT may provide effective regional control in this patient group.

The omission of ALND in patients with micrometastatic SLNs was shown to be an acceptable treatment strategy based on the 10 year follow-up results of the IBCSG 23-01 trial.^12^ The authors reported that survival outcomes in patients who did not undergo axillary dissection were non-inferior to those of ALND. The IBCSG 23-01 trial included patients who underwent a mastectomy, although no locoregional radiotherapy was administered according to their protocol. In the present study, we included only patients with macrometastasis in the SLN group. Based on our findings, the results and implications of IBCSG 23-01 may also be applicable to patients who present with a macrometastatic axillary disease burden.

Less extensive axillary surgery may raise a concern for possible remnant axillary nodal disease, potentially resulting in subsequent regional recurrence. In addition to a positive SLN, additional metastatic non-SLNs have been reported in 33–35% of patients who underwent ALND.^13^ In the present study, we observed no significant differences in recurrence and survival rates between the SLNB-alone and ALND groups of patients with cT1-2, cN0 disease with one to two positive SLNs who underwent TM.

It is well known that adjuvant RT plays a major role in locoregional control in patients with early breast cancer who undergo breast-conserving surgery and TM, especially in patients with positive metastatic lymph nodes. In a meta-analysis of 22 trials performed by the Early Breast Cancer Trialists’ Collaborative Group (EBCTCG), post-mastectomy radiotherapy, in addition to ALND, was found to reduce both locoregional and overall recurrence in patients with one to three positive nodes.^14^ According to the recently updated AMAROS trial, after a median follow-up of 10 years and including 17% of their study cohort who underwent TM, both SLNB with axillary radiation and ALND provided a low axillary recurrence rate without substantial differences in OS, DFS, and locoregional control in patients with cT1-2 cN0 SLN-positive breast cancer.^6^ In the updated AMAROS trial analysis, the 10 year cumulative incidence of axillary recurrence was 0.93% after ALND and 1.82% after RT (HR 1.71, 95% CI 0.18–1.68), with no notable differences in OS and DFS. However, clinical signs of arm lymphedema were substantially reduced in the axillary radiotherapy group than those in the ALND group. SLNB combined with axillary RT provided oncologic outcomes comparable with those of ALND while improving quality of life and reducing the risk of developing lymphedema. The results of the prospective randomized controlled SUPREMO trial will be available in the near future and may provide further evidence for the role of post-mastectomy radiotherapy in pN1 patients with SLNB alone or ALND.^15^

Furthermore, appropriate adjuvant systemic treatment may impact a role in locoregional control. In the present study, 76 and 86% of our patients received chemotherapy and endocrine treatment, respectively; these proportions are higher than the 61 and 77% of patients who received chemotherapy and hormonal therapy, respectively, in the AMAROS trial. Adjuvant radiation was provided to only 30.4 and 26.6% of patients in the SLNB-alone and ALND groups, respectively, based on individualized clinical decisions. Our results indicate that adjuvant radiation may provide benefits in terms of effective locoregional control in SLN-positive patients undergoing TM and SLNB alone.

Nevertheless, the limitations of the present study need to be addressed. Along with the retrospective nature of the study, there could be a potential selection bias in terms of eligible patients who underwent SLNB, who might have a more favorable initial disease status than those who underwent ALND. Furthermore, within the SLNB cohort, we admit that lower nodal burden could have potentially influenced the clinical decision of omitting adjuvant radiation. Next, the proportion of patients with three positive SLNs was relatively low; hence, the generalizability of the present results should be cautiously interpreted in this subgroup. It may be more suitable to apply the results to the breast cancer patients who have only one to two metastatic SLNs. In addition, details regarding RT tended to vary, given that RT was performed at two different institutions with different treatment strategies, and the treatment period included the time frame of transition to hypofractionation for radiation delivery.

## Conclusion

In patients with early breast cancer with no palpable axillary lymphadenopathy who are candidates for TM and have a limited number of positive SLNs, *less invasive axillary surgery* may be a feasible option without compromising disease recurrence or survival. *Adjuvant radiation* may allow better locoregional control in patients undergoing less extensive axillary surgery and TM.

### Supplementary Information

Below is the link to the electronic supplementary material.

Supplementary file 1 (DOCX 124 kb)

## Data Availability

The datasets generated and/or analyzed during the current study are not publicly available due to the inclusion of personal information but are available from the corresponding author on reasonable request after adequate processing.
