# Supplementary material for: Oncological Safety of Skipping Axillary Lymph Node Dissection in Patients with Clinical N0, Sentinel Node-Positive Breast Cancer Undergoing Total Mastectomy
Source: Ann Surg Oncol. 2024 Feb 17;31(5):3168–76. doi: 10.1245/s10434-024-15049-7 (PMC10997532; doi:10.1245/s10434-024-15049-7)

**Supplements**

**Oncological safety of skipping axillary lymph node dissection in patients with clinical N0,** **sentinel node-positive breast cancer undergoing total mastectomy**

Jung Whan Chun^1^, Eunhye Kang^1^, Hong-Kyu Kim^1^, Han-Byoel Lee^1^, Hyeong-Gon Moon^1^, Jong Won Lee^2*^ and Wonshik Han^1*^

^1^Division of Breast Surgery, Department of Surgery, Seoul National University College of Medicine, Seoul, Republic of Korea

^2^Division of Breast Surgery, Department of Surgery, University of Ulsan College of Medicine, Asan Medical Center, Seoul, Republic of Korea.

^*^Corresponding authors:

Wonshik Han MD, PhD

Department of Surgery and Cancer Research Institute, Seoul National University College of Medicine, 101 Daehak-ro, Jongno-gu, Seoul 110-774, Republic of Korea; Fax: 82-2-766-3975; E-mail: hanw@snu.ac.kr

Jong Won Lee, MD, PhD

Division of Breast Surgery, Department of Surgery, University of Ulsan College of Medicine, Asan Medical Center, 88, Olympic-ro 43-gil, Songpa-Gu, Seoul 05505, Republic of Korea. Tel: 82-2-3010-1729; Fax: 82-2-474-9027; E-mail: [jjjongwr@hanmail.net](mailto:jjjongwr@hanmail.net)

**Supplemental material**

**Supplemental Tables**

**eTable 1.** Baseline clinicopathologic characteristics of patients who underwent SLNB alone or ALND. ALND, axillary lymph node dissection; SLNB, sentinel lymph node biopsy.

**eTable 2.** Within the matched cohort**s,** clinicopathologic characteristics of patients who received RT according to the axillary surgery (SLNB-alone *vs.* ALND). ALND, axillary lymph node dissection; RT, radiation therapy; SLNB, sentinel lymph node biopsy.

**eTable 3.** Within the matched cohorts, clinicopathologic characteristics of patients without RT according to the axillary surgery extent (SLNB-alone *vs.* ALND). ALND, axillary lymph node dissection; RT, radiation therapy; SLNB, sentinel lymph node biopsy.

**Supplemental figure**

**eFigure 1.** Within the matched cohorts**,** The five-year locoregional recurrence-free survival and distant metastasis-free survival of patients who underwent ALND.

eTable 1. Baseline clinicopathologic characteristics of patients who underwent SLNB-alone or ALND.

|  | **SLNB only** | **ALND** | **p-value** |
| --- | --- | --- | --- |
| **Number of patients** | 237 | 406 |  |
|  |  |  |  |
| **Age (years)** | 52.9 ± 11.6 | 50.8 ± 10.5 | 0.02 |
|  |  |  | 0.08 |
| ≤ 50 | 107 (45.1) | 212 (52.2) |  |
| > 50 | 130 (54.9) | 194 (47.8) |  |
|  |  |  |  |
| **Follow-up period (month)** | 63.9 ± 18.6 | 65.7 ± 19.4 | 0.24 |
|  |  |  |  |
| **Clinical T stage** |  |  | 0.2 |
| 1 | 57 (24.1) | 85 (20.9) |  |
| 2 | 145 (61.2) | 239 (58.9) |  |
| 3 | 35 (14.8) | 82 (20.2) |  |
|  |  |  |  |
| **Number of metastatic sentinel node(s)** |  |  | <0.001 |
| 1 | 208 (87.8) | 274 (67.5) |  |
| 2 | 26 (11.0) | 117 (28.8) |  |
| 3 | 3 (1.3) | 15 (3.7) |  |
|  |  |  |  |
| **Number of total axillary nodes** |  |  | <0.001 |
|  | 7.9 ± 5.2 | 18.0 ± 6.1 |  |
|  |  |  |  |
| **Number of total metastatic node(s)** |  |  | <0.001 |
|  | 1.2 ± 0.6 | 2.0 ± 1.6 |  |
|  |  |  |  |
| **Pathologic T stage** |  |  | 0.17 |
| 1 | 88 (37.1) | 131 (32.3) |  |
| 2 | 139 (58.6) | 241 (59.4) |  |
| 3 | 9 (3.8) | 29 (7.1) |  |
| 4 | 1 (0.4) | 5 (1.2) |  |
|  |  |  |  |
| **Pathologic N stage** |  |  | <0.001 |
| 1 | 232 (97.9) | 358 (88.2) |  |
| 2 | 3 (1.3) | 41 (10.1) |  |
| 3 | 2 (0.8) | 7 (1.7) |  |
|  |  |  |  |
| **Biologic subtype** |  |  | 0.46 |
| ER+/ HER2- | 181 (76.4) | 290 (71.4) |  |
| ER+/ HER2+ | 27 (11.4) | 53 (13.1) |  |
| ER-/ HER2+ | 14 (5.9) | 36 (8.9) |  |
| ER-/ HER2- | 15 (6.3) | 27 (6.7) |  |
|  |  |  |  |
| **Histologic grade** |  |  | 0.18 |
| 1 | 14 (5.9) | 29 (7.1) |  |
| 2 | 168 (70.9) | 259 (63.8) |  |
| 3 | 55 (23.2) | 118 (29.1) |  |
|  |  |  |  |
| **Lymphovascular invasion** |  |  | 0.004 |
| Yes | 105 (44.3) | 228 (56.2) |  |
| No | 132 (55.7) | 178 (43.8) |  |
|  |  |  |  |
| **Adjuvant chemotherapy** |  |  | <0.001 |
| Yes | 182 (76.8) | 355 (87.4) |  |
| No | 55 (23.2) | 51 (12.6) |  |
|  |  |  |  |
| **Adjuvant radiation** |  |  | 0.28 |
| Yes | 72 (30.4) | 140 (34.5) |  |
| No | 165 (69.6) | 266 (65.5) |  |
|  |  |  |  |
| **Adjuvant endocrine therapy** |  |  | 0.27 |
| Yes | 205 (86.5) | 338 (83.3) |  |
| No | 32 (13.5) | 68 (16.7) |  |
|  |  |  |  |
| **Recurrence** |  |  |  |
| **Locoregional recurrence** |  |  | 0.66 |
| Yes | 11 (4.6) | 16 (3.9) |  |
| No | 226 (95.4) | 390 (96.1) |  |
| **Distant metastasis** |  |  | 0.34 |
| Yes | 15 (6.3) | 34 (8.4) |  |
| No | 222 (93.7) | 372 (91.6) |  |

ALND, axillary lymph node dissection; SLNB, sentinel lymph node biopsy.

eTable 2. Within the matched cohorts, clinicopathologic characteristics of patients who received RT according to the axillary surgery (SLNB-alone *vs.* ALND).

|  | **SNB alone** | **ALND** | **p-value** |
| --- | --- | --- | --- |
| **Number of patients** | 72 | 63 |  |
|  |  |  |  |
| **Age (years)** | 51.9 ± 10.3 | 51.7 ± 8.8 | 0.92 |
|  |  |  | 0.74 |
| ≤ 50 | 34 (47.2) | 28 (44.4) |  |
| > 50 | 38 (52.8) | 35 (55.6) |  |
|  |  |  |  |
| **Follow-up period (month)** | 61.9 ± 16.6 | 65.4 ± 19.3 | 0.27 |
|  |  |  |  |
| **Clinical T stage** |  |  | 0.73 |
| 1 | 12 (16.7) | 13 (20.6) |  |
| 2 | 48 (66.7) | 38 (60.3) |  |
| 3 | 12 (16.7) | 12 (19.0) |  |
|  |  |  |  |
| **Number of metastatic Sentinel node(s)** |  |  | 0.4 |
| 1 | 62 (86.1) | 52 (82.5) |  |
| 2 | 7 (9.7) | 10 (15.9) |  |
| 3 | 3 (4.2) | 1 (1.6) |  |
|  |  |  |  |
| **Number of total axillary nodes** |  |  | <0.001 |
|  | 7.1 ± 4.6 | 19.2 ± 6.3 |  |
|  |  |  |  |
| **Number of total metastatic node(s)** |  |  | <0.001 |
|  | 1.3 ± 0.8 | 2.6 ± 2.2 |  |
|  |  |  |  |
| **Pathologic T stage** |  |  | 0.18 |
| 1 | 15 (20.8) | 9 (14.3) |  |
| 2 | 48 (66.7) | 37 (58.7) |  |
| 3 | 8 (11.1) | 15 (23.8) |  |
| 4 | 1 (1.4) | 2 (3.2) |  |
|  |  |  |  |
| **Pathologic N stage** |  |  | 0.02 |
| 1 | 67 (93.1) | 50 (79.4) |  |
| 2 | 3 (4.2) | 12 (19.0) |  |
| 3 | 2 (2.8) | 1 (1.6) |  |
|  |  |  |  |
| **Biologic subtype** |  |  | 0.11 |
| ER+/ HER2- | 55 (76.4) | 36 (57.1) |  |
| ER+/ HER2+ | 7 (9.7) | 10 (15.9) |  |
| ER-/ HER2+ | 5 (6.9) | 10 (15.9) |  |
| ER-/ HER2- | 5 (6.9) | 7 (11.1) |  |
|  |  |  |  |
| **Histologic grade** |  |  | 0.77 |
| 1 | 4 (5.6) | 2 (3.2) |  |
| 2 | 46 (63.9) | 40 (63.5) |  |
| 3 | 22 (30.6) | 21 (33.3) |  |
|  |  |  |  |
| **Lymphovascular invasion** |  |  | 0.26 |
| Yes | 33 (45.8) | 35 (55.6) |  |
| No | 39 (54.2) | 28 (44.4) |  |
|  |  |  |  |
| **Adjuvant chemotherapy** |  |  | 0.002 |
| Yes | 59 (81.9) | 62 (98.4) |  |
| No | 13 (18.1) | 1 (1.6) |  |
|  |  |  |  |
| **Adjuvant endocrine therapy** |  |  | 0.09 |
| Yes | 62 (86.1) | 47 (74.6) |  |
| No | 10 (13.9) | 16 (25.4) |  |
|  |  |  |  |
| **Recurrence** |  |  |  |
| **regional recurrence** |  |  | NA |
| Yes | 0 (0) | 0 (0) | D |
| No | 72 (100) | 63 (100) |  |
| **Distant metastasis** |  |  | 0.56 |
| Yes | 3 (4.2) | 4 (6.3) |  |
| No | 69 (95.8) | 59 (93.7) |  |

ALND, axillary node dissection; RT, radiation therapy; SLNB, sentinel lymph node biopsy.

eTable 3. Within the matched cohorts, clinicopathologic characteristics of patients without RT according to the axillary surgery extent (SLNB-alone *vs.* ALND).

|  | **SNB alone** | **ALND** | **p-value** |
| --- | --- | --- | --- |
| **Number of patients** | 165 | 174 |  |
|  |  |  |  |
| **Age (years)** | 53.3 ± 12.2 | 51.5 ± 9.8 | 0.13 |
|  |  |  | 0.45 |
| ≤ 50 | 73 (44.2) | 84 (48.3) |  |
| > 50 | 92 (55.8) | 90 (51.7) |  |
|  |  |  |  |
| **Follow-up period (month)** | 64.7 ± 19.4 | 65.1 ± 17.9 | 0.85 |
|  |  |  |  |
| **Clinical T stage** |  |  | 1 |
| 1 | 45 (27.3) | 44 (25.3) |  |
| 2 | 97 (55.8) | 107 (61.5) |  |
| 3 | 23 (13.9) | 23 (13.2) |  |
|  |  |  |  |
| **Number of metastatic Sentinel node(s)** |  |  | 1 |
| 1 | 208 (87.8) | 208 (87.8) |  |
| 2 | 26 (11.0) | 26 (11.0) |  |
| 3 | 3 (1.3) | 3 (1.3) |  |
|  |  |  |  |
| **Number of total axillary nodes** |  |  | <0.001 |
|  | 8.3 ± 5.4 | 16.5 ± 5.5 |  |
|  |  |  |  |
| **Number of total metastatic node(s)** |  |  | <0.001 |
|  | 1.1 ± 0.4 | 1.3 ± 0.6 |  |
|  |  |  |  |
| **Pathologic T stage** |  |  | 0.48 |
| 1 | 73 (44.2) | 66 (37.9) |  |
| 2 | 91 (55.2) | 105 (60.3) |  |
| 3 | 1 (0.6) | 2 (1.1) |  |
| 4 | 0 (0.0) | 1 (0.6) |  |
|  |  |  |  |
| **Pathologic N stage** |  |  | 0.32 |
| 1 | 165 (100.0) | 173 (99.4) |  |
| 2 | 0 (0.0) | 1 (0.6) |  |
| 3 | 0 (0.0) | 0 (0.0) |  |
|  |  |  |  |
| **Biologic subtype** |  |  | 0.64 |
| ER+/ HER2- | 126 (76.4) | 132 (75.9) |  |
| ER+/ HER2+ | 20 (12.1) | 19 (10.9) |  |
| ER-/ HER2+ | 9 (5.5) | 15 (8.6) |  |
| ER-/ HER2- | 10 (6.1) | 8 (4.6) |  |
|  |  |  |  |
| **Histologic grade** |  |  | 0.04 |
| 1 | 10 (6.1) | 18 (10.3) |  |
| 2 | 122 (73.9) | 107 (61.5) |  |
| 3 | 33 (20.0) | 49 (28.2) |  |
|  |  |  |  |
| **Lymphovascular invasion** |  |  | 0.05 |
| Yes | 72 (43.6) | 94 (54.0) |  |
| No | 93 (56.4) | 80 (46.0) |  |
|  |  |  |  |
| **Adjuvant chemotherapy** |  |  | 0.01 |
| Yes | 123 (74.5) | 148 (85.1) |  |
| No | 42 (25.5) | 26 (14.9) |  |
|  |  |  |  |
| **Adjuvant endocrine therapy** |  |  | 0.67 |
| Yes | 143 (86.7) | 148 (85.1) |  |
| No | 22 (13.3) | 26 (14.9) |  |
|  |  |  |  |
| **Locorecurrence** |  |  |  |
| **regional recurrence** |  |  | 0.17 |
| Yes | 11 (6.7) | 6 (3.4) |  |
| No | 154 (93.3) | 168 (96.6) |  |
| **Distant metastasis** |  |  | 0.72 |
| Yes | 12 (7.3) | 11 (6.3) |  |
| No | 153 (92.7) | 163 (93.7) |  |

ALND, axillary node dissection; RT, radiation therapy; SLNB, sentinel lymph node biopsy.

**eFigure 1. The five-year locoregional recurrence-free survival and distant metastasis-free survival of patients who underwent ALND within the matched cohorts.** The five-year locoregional recurrence-free survival (LRRFS) (a.) and distant metastasis-free survival (DMFS) (b.) were compared among patients who underwent ALND according to whether or not they received radiation therapy. (RTx=1: with radiation therapy, RTx=2: without radiation therapy). ALND, axillary node dissection.

a. b.


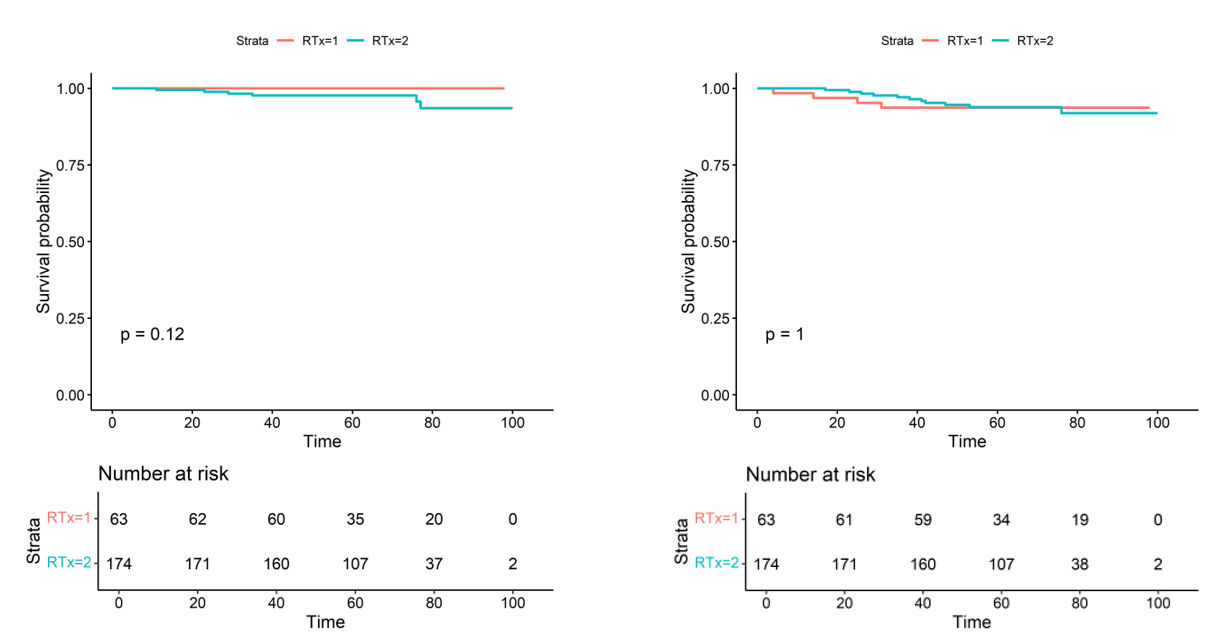

Supplement: Supplementary file 1 — (DOCX 124 kb) [file 10434_2024_15049_MOESM1_ESM.docx]
